# The Psychometric Properties and Clinical Utility of the Korean Version of GAD-7 and GAD-2

**DOI:** 10.3389/fpsyt.2019.00127

**Published:** 2019-03-18

**Authors:** Jung-Kwang Ahn, Yeseul Kim, Kee-Hong Choi

**Affiliations:** ^1^Department of Psychology, Korea University Mind Health Institute, Korea University, Seoul, South Korea; ^2^Department of Psychology, Korea University, Seoul, South Korea

**Keywords:** generalized anxiety disorder, GAD-7, GAD-2, screening instruments, sensitivity, specificity

## Abstract

Generalized anxiety disorder (GAD) is a common but serious form of anxiety disorder. Despite this, the rate of GAD recognition in primary care remains low in both Western and Eastern countries. The GAD-7 and GAD-2 were developed to efficiently identify people with GAD, and their reliability and validity have been well-documented in Western countries. The GAD-7 and GAD-2 have also been widely utilized to screen for other anxiety disorders; however, their diagnostic utility has not been fully justified with empirical support, especially in East Asian samples. In this study, we examined the diagnostic sensitivity and specificity of these screening tools for identifying individuals with GAD or other anxiety disorders, and recommended screening cutoff scores for GAD and other anxiety disorders for use in Korea. Based on the rigorous standard suggested by the Quality Assessment of Diagnostic Accuracy Studies-2, a total of 1,157 participants randomly recruited from the community completed the GAD-7, GAD-2, and other anxiety and depression measures in a counter-balanced order. All participants were assessed, and their psychiatric diagnosis confirmed through a structured clinical interview conducted by independent clinicians blinded to the results of the self-report questionnaires. The GAD-7 and GAD-2 both showed excellent reliability and validity. Notably, both the GAD-7 and GAD-2 demonstrated acceptable diagnostic accuracy in detecting GAD with similar recommended cut-off scores as those reported in Western countries, but unacceptable diagnostic accuracy for other anxiety disorders. We conclude that given their brevity, the GAD-7 and GAD-2 can be well-utilized to identify people with GAD for preventative evaluation and treatment in Korea. Use of the GAD-7 and GAD-2 for screening other anxiety disorders should be cautioned.

## Introduction

Generalized anxiety disorder (GAD) is one of the most common yet serious forms of anxiety disorder, characterized mainly by pervasive, uncontrollable, and long-lasting worries. According to a global review on the prevalence of anxiety disorders, the lifetime prevalence of GAD was estimated to be 6.2% (95% confidence interval [CI]: 4.0–9.2%) (1) and 2.2% among adolescents (2). GAD often follows a chronic course and deteriorates overall quality of life and subjective well-being (3–5). Given the chronic nature and adverse functional outcomes of GAD, early diagnosis and timely intervention are essential for individuals with GAD. However, due to frequent comorbidities and the nature of the disease, which is accompanied by various physical symptoms, approximately half of individuals with GAD consulted their primary care physicians rather than mental health professionals when seeking treatment for anxiety symptoms (6). Unfortunately, the rate of GAD recognition in primary care remains between 29.0% and 34.4% in Western countries (6, 7) and at 33.3% in non-Western countries (8, 9). Given this, a valid and reliable diagnostic tool for GAD in a brief format (i.e., a minimum number of questions) would facilitate early detection and proper timely intervention, not only in primary care institutions but in mental health settings as well.

The generalized anxiety disorder 7-item scale [GAD-7; (10)] was developed with the clear purpose of screening patients with GAD. The scale has also been widely used in both clinical and research settings to monitor the severity of GAD symptoms. It was proven to be a reliable and valid instrument, and its seven items reflect most of the GAD diagnostic domains in the *Diagnostic and Statistical Manual of Mental Disorders, 4*^*th*^ *Edition* (DSM-IV) (11). Further, the GAD-7 has been found to have clinical utility in screening for other anxiety disorders in several studies, although its sensitivity and specificity were lower than for GAD (12, 13). GAD is highly comorbid with other anxiety disorders and typically precedes the onset of the comorbidities, which contributed to the conceptualization of GAD as the “basic” anxiety disorder (14, 15). In sum, as GAD also shares common features of other anxiety disorders including uncontrollable worry and accompanying somatic symptoms (16), a screening tool for GAD may potentially detect other anxiety disorders as well.

A diagnostic meta-analysis of the GAD-7 reported its sensitivity and specificity for screening GAD as 0.83 (95% CI: 0.71–0.91) and 0.84 (95% CI: 0.70–0.92), respectively, at the cut-point of 8 or greater (17). For identifying anxiety disorders, sensitivity and specificity values ranged from 0.77 to 0.91 and 0.74 to 0.83, respectively, at the same cut-point (17). However, to use the GAD-7 as a screening tool for anxiety disorders, the cutoff score should be studied further (17).

Among its seven items of the GAD-7, items 1 and 2 represent the core anxiety symptoms. These thus comprise the GAD-2, an ultra-brief version of the GAD-7 questionnaire, which can be used in primary care settings with limited time and resources (12). Plummer et al. (17) reported acceptable sensitivity and specificity values for screening GAD at a GAD-2 cut-point of 3 [sensitivity: 0.76 [95% CI: 0.55–0.89], specificity: 0.81 [95% CI: 0.60–0.92]]. However, empirical evidence for GAD-2 is also insufficient to determine a cutoff score for identifying any other anxiety disorders, because sensitivity and specificity values were varied (17).

Therefore, the purposes of this study were (1) to examine the psychometric properties and diagnostic sensitivity and specificity of these screening tools for identifying individuals with any anxiety disorder, and (2) to determine cutoff scores for identifying both GAD and other anxiety disorders.

## Method

### Participants

The present study was carried out as part of a large nationally funded research project entitled, the Development and Validation of the Korean Depression and Anxiety Scales, conducted from September 2015 to August 2018. The ethical approval was accepted by Korea University Institutional Review Board. A total of 1,228 individuals were recruited for this study through two routes: online recruiting advertisements and introduction to potential research participants by hospital staff. All individuals voluntarily opted to participate in the study. The only inclusion criterion was being age of 19 years or older. Exclusion criteria were not specified to minimize sampling bias. For rigorous evaluation of the accuracy of the screening tools, the methodology of the Quality Assessment of Diagnostic Accuracy Studies-2 (QUADAS-2) (18) was applied. The QUADAS-2 evaluates the quality of screening tools in four domains: patient selection, index test, reference standard, and flow and timing. To avoid bias in participant selection, the samples in this study were randomly recruited and minimal exclusion criteria were specified. However, 71 individuals (5.78%) were excluded either because they did not complete the questionnaire or because they could not answer questions properly as a result of their psychiatric or medical symptoms. A final total of 1,157 participants were included in the present analysis.

### Measures

#### Mini-International Neuropsychiatric Interview-Plus Version 5.0.0 (MINI)

The MINI is a structured clinical interview used to diagnose psychiatric disorders according to the DSM-IV and the *International Classification of Diseases, 10th Edition* (ICD-10). The Korean version of the MINI (19), which showed overall good agreement between MINI based and expert diagnoses, was used in this study. The MINI was utilized as a reference standard (i.e., criterion). The one-on-one, in-person clinical diagnostic interview took ~30–50 min per participant. The MINI was administered by licensed clinical psychologists, psychiatrists and supervised clinical psychology senior students. The inter-rater reliability of the MINI was 0.92. Final psychiatric diagnoses were confirmed by licensed clinical psychologists and the psychiatrist.

#### GAD-7 and GAD-2

The GAD-7 is a simple, 7-item self-administered instrument designed to screen for GAD and used to assess the intensity of symptoms. Subjects are asked to rate the frequency at which they have been disturbed by each symptom over the past 2 weeks using a 4-point Likert scale. The Korean version of the GAD-7 (20), which is available on the Patient Health Questionnaire website (http://www.phqscreeners.com), was used in the present study. In the previous research (21), the items of the Korean version of the GAD-7 were translated and then back-translated by an independent bilingual speaker. The original version and back translated versions were compared by another native English speaker who concluded that both were identical. Korean version of GAD-7 showed excellent internal consistency (α = 0.93).

The first three items of the GAD-7 relate to two core criteria of GAD (A and B) defined in the DSM-IV (10, 11). Therefore, use of a short-form version consisting of only the first two items was proposed, resulting in the GAD-2 scale. The GAD-2 is reported to be a reliable and valid tool for screening GAD, both when performed alone or when extracted from previous GAD-7 results (22). The two items showed the highest correlation with the GAD-7 total score (Pearson's *r* = 0.94, *p* < 0.01).

#### Anxiety Measures

##### Beck Anxiety Inventory (BAI)

The BAI (23) scale is widely used to assess the severity of anxiety and track treatment progress. This 21-item self-report inventory covers the affective, cognitive, and physical domains of anxiety. The measure asks respondents to indicate the extent to which they have suffered from each symptom over the past week using a 4-point Likert scale. The Korean version of the BAI (24) was used in this study, and showed excellent internal consistency (α = 0.96).

##### Penn State Worry Questionnaire (PSWQ)

The PSWQ (25) is a 16-item self-administered instrument designed to measure the frequency and intensity of pathological worry. Each item is assessed on a 5-point Likert scale. In this study, the Korean version of the scale (26) was used, and showed very good internal consistency (α = 0.85).

#### Depression Measures

##### Beck Depression Inventory-II (BDI-II)

The BDI-II (27) is a well-accepted self-report inventory consisting of 21 items that assess the affective, cognitive, motivational, and physiological severity of depressive symptoms. Subjects rate each item using a 4-point Likert scale. The Korean version of the BDI-II (28) was used in this study, and showed excellent internal consistency (α = 0.95).

##### Center for Epidemiologic Studies Depression Scale (CES-D)

The CES-D (29) is a 20-item self-report measure developed to easily identify depression in the general population. Subjects are asked to indicate how often they have experienced emotional and physical symptoms and interpersonal difficulties over the previous 7 days. Each item is rated on a 4-point Likert scale. In the present study, the Korean version of the CES-D (30) was used, and showed very good internal consistency (α = 0.85).

### Research Design

When individuals indicated their intention to participate verbally or by the response to an e-mail, research assistants coordinated their dates for participation. Participants were invited to a University research lab or two other general hospitals and received a detailed explanation of the current study. After obtaining a signed written informed consent from each participant, they were asked to complete a self-report assessment battery consisting of a demographic information questionnaire, the GAD-7, and other anxiety or depression measures. In most cases, the questionnaires were immediately retrieved, but for some participants who needed additional time for completion, the remaining items were completed at home and returned within a week at the latest. Licensed clinical psychologists, psychiatrists, and trained and supervised clinical psychology graduate research assistants administered face-to-face diagnostic interviews using the MINI (31) before or after participants completing the self-report assessment battery. All procedures, including the questionnaires and interview, took approximately 45–75 min. Participants were compensated for their participation, as specified in the approved Institutional Review Boards protocol. According to the recommendation of the QUADAS-2, to avoid bias in sampling and evaluation, all participants were treated the same way regardless of patient or non-patient. The self-report assessment battery and the MINI were conducted, scored, and interpreted separately by independent evaluators without knowing the results of the assessment battery or psychiatric diagnoses from the MINI.

### Analysis

The internal consistency of responses in the GAD-7 was examined using Cronbach's alpha and item-total correlation. Validity evidence was collected not from a single source but from several, following the recommendations of the *Standards for Educational and Psychological Testing* provided by AERA, APA, and NCME (32). Convergent validity was assessed by calculating correlations of the GAD-7 and GAD-2 with other anxiety scales, namely the BAI and PSWQ. Discriminant validity was assessed by examining correlations of the GAD-7 and GAD-2 with depression measures, namely the BDI-II and CES-D. Discriminant validity was also assessed by independent *t*-test. The mean scores of the GAD-7 and GAD-2 in participants with GAD were compared to the scores of the individuals without GAD. To avoid multiple comparison problems, we use Bonferroni correction, and the *p*-value was 0.0125 in these independent *t*-tests. The examination of diagnostic criterion validity included receiver operating characteristic (ROC) analyses and investigation of diagnostic sensitivity and specificity, positive and negative predictive values (PPV and NPV), and positive and negative likelihood ratios (PLR and NLR) at various cutoff scores concerning the diagnosis of GAD or any anxiety disorder based on the MINI. The optimal cutoff points for the GAD-7 and GAD-2 were determined where both diagnostic sensitivity and specificity were maximized. Data analysis using the Statistical Package for the Social Sciences (SPSS) version 24.

## Results

### Demographics

The total sample (*N* = 1,157) had a mean age of 37.31 (*SD* = 14.76, range 19–85), and 772 (66.7%) of the subjects were women. The mean years of education was 14.63 (*SD* = 2.98). All participants were South Korean.

Of the 1,157 participants, 90 (7.7%) met the DSM-IV criteria for current GAD, and only 15 (1.3%) were GAD only. Additionally, 128 (11.1%) met the criteria for any current anxiety disorder without GAD, 132 (11.4%) for any depressive disorder without GAD, and 56 (4.8%) for MDD without GAD. A total of 684 (59.1%) participants did not meet the DSM-IV criteria for any mental disorders (Table 1).

**Table 1:** Baseline characteristics.

**Characteristics**	**GAD only**	**GAD**	**AD wo GAD**	**DD wo GAD**	**MDD wo GAD**	**No mental disorder**
	**(*n* = 15)**	**(*n* = 90)**	**(*n* = 128)**	**(*n* = 132)**	**(*n* = 56)**	**(*n* = 684)**
	***M (SD)***	***M (SD)***	***M (SD)***	***M (SD)***	***M (SD)***	***M (SD)***
Age *(SD)* [range]	34.87 (11.74) [22–61]	39.65 (14.37) [19–80]	39.60 (15.09) [19–82]	43.78 (17.33) [19–82]	42.63 (16.01) [20–78]	35.72 (13.87) [19–85]
Women (%)	12 (80.0)	65 (72.2)	85 (66.4)	85 (64.4)	35 (62.5)	455 (66.6)
Education *(SD)*	15.13 (2.00)	13.63 (3.28)	13.73 (2.98)	13.09 (3.32)	13.19 (3.75)	15.07 (2.77)
GAD-7 *(SD)*	10.07 (5.74)	13.29 (5.43)	6.66 (5.52)	7.88 (5.56)	10.13 (5.51)	2.34 (2.96)
GAD-2 *(SD)*	3.27 (1.75)	4.01 (1.73)	2.06 (1.83)	2.33 (1.82)	3.07 (1.81)	0.68 (1.02)
BAI *(SD)*	14.67 (7.17)	26.03 (14.89)	14.27 (13.08)	17.02 (12.60)	20.64 (11.75)	4.22 (5.29)
PSWQ *(SD)*	61.29 (12.31)	65.25 (10.76)	52.55 (13.57)	53.16 (12.81)	56.85 (12.03)	41.82 (11.02)
BDI-II *(SD)*	23.27 (8.35)	32.11 (12.43)	20.89 (12.93)	23.85 (12.52)	29.04 (11.35)	8.71 (7.13)
CES-D *(SD)*	28.67 (12.02)	37.54 (12.14)	24.20 (13.32)	27.22 (11.87)	33.96 (9.67)	12.43 (7.98)

*GAD only, generalized anxiety disorder without the comorbid disorder(s); GAD, generalized anxiety disorder with or without the comorbid disorder(s); AD wo GAD, anxiety disorder without general anxiety disorder; DD wo GAD, any depressive disorder without general anxiety disorder; MDD wo GAD, major depressive disorder without general anxiety disorder*.

### Reliability and Divergent Validity

Cronbach's α for the GAD-7 was 0.93, indicating excellent internal consistency in this study sample. The GAD-7 score was well-correlated with other anxiety measures: BAI score, *r* = 0.78 (95% CI:0.74–0.81), *p* < 0.001, PSWQ score, *r* = 0.72 (95% CI:0.68–0.75), *p* < 0.001. The GAD-7 score was also significantly correlated with the depression scales: BDI score, *r* = 0.80 (95% CI:0.77–0.83), *p* < 0.001; CES-D score, *r* = 0.83 (95% CI:0.80–0.85), *p* < 0.001.

Cronbach's α for the GAD-2 was 0.86, also indicating excellent internal consistency. The GAD-2 score was well-correlated with the BAI score, *r* = 0.72 (95% CI:0.68–0.76), *p* < 0.001, and the PSWQ score, *r* = 0.68 (95% CI:0.65–0.72), *p* < 0.001. The GAD-2 score was also significantly correlated with the depression scales: BDI score, *r* = 0.74 (95% CI:0.70–0.77), *p* < 0.001; CES-D score, *r* = 0.79 (95% CI:0.76–0.81), *p* < 0.001.

Table 1 shows the means and standard deviations of the GAD-7 and GAD-2, other anxiety scales, and depression scales for the distinct psychiatric diagnoses groups. Independent *t*-test showed that subjects with GAD had significantly higher means on the GAD-7 and GAD-2 than those without GAD [i.e., GAD vs. other anxiety disorders without GAD on GAD-7, [*t*_(216)_ = 8.80, *p* < 0.001]; GAD vs. other anxiety disorders without GAD on GAD-2, [*t*_(216)_ = 7.91, *p* < 0.001]; GAD vs. depressive disorders without GAD on GAD-7, [*t*_(220)_ = 7.18, *p* < 0.001]; GAD vs. depressive disorders without GAD on GAD-2, [*t*_(220)_ = 6.91, *p* < 0.001]; GAD vs. MDD without GAD on GAD-7, [*t*_(144)_ = 3.40, *p* = 0.001]; GAD vs. MDD without GAD on GAD-2, [*t*_(144)_ = 3.14, *p* = 0.002]; GAD vs. no mental disorders on GAD-7, [*t*_(770)_ = 29.22, *p* < 0.001]; GAD vs. no mental disorders on GAD-2, [*t*_(770)_ = 26.49, *p* < 0.001]].

### Accuracy of the GAD-7 and GAD-2

**Figure 1:**
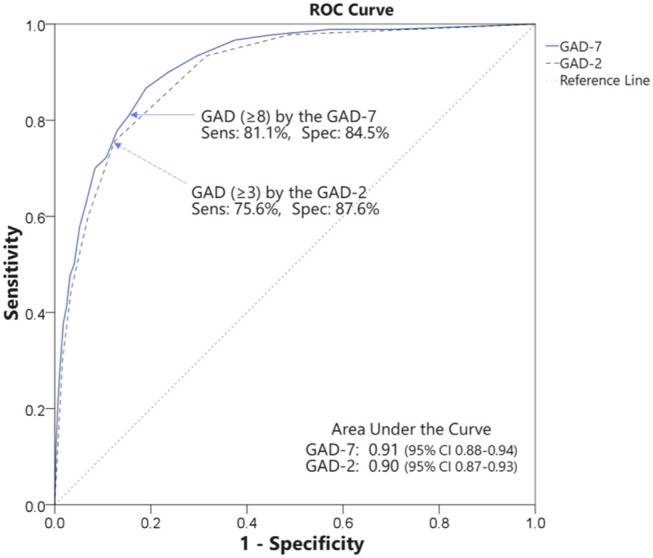
ROC curves for detection of GAD by GAD-7 and GAD-2.

ROC analyses were conducted to examine the accuracy of the GAD-7 and GAD-2 questionnaires in identifying GAD or any anxiety disorder. The ROC curves are illustrated in Figure 1.

ROC analysis of the GAD-7 for identifying GAD exhibited an area under the curve (AUC) of 0.91 (95% CI: 0.88–0.94, SE = 0.02, *p* < 0.001), indicating high accuracy (33). At a cutoff score of 8 or greater, the balance of sensitivity and specificity reached its maximum. Sensitivity was 0.81 (95% CI: 0.72–0.88) and specificity was 0.85 (95% CI: 0.84–0.85) with a PPV of 0.31 (95% CI: 0.25–0.37), an NPV of 0.98 (95% CI: 0.97–0.99), a PLR of 5.25 (95% CI: 4.42–5.93), and an NLR of 0.22 (95% CI: 0.14–0.34) (Table 2).

**Table 2:** Diagnostic sensitivity, specificity, positive and negative predictive value, and positive and negative likelihood ratio of GAD-7 and GAD-2.

	**Cutoff score**	**Sensitivity (95% CI)**	**Specificity (95% CI)**	**PPV (95% CI)**	**NPV (95% CI)**	**PLR (95% CI)**	**NLR (95% CI)**
**GAD-7**							
GAD	≥5	0.933 (0.857–0.972)	0.706 (0.699–0.709)	0.211 (0.171–0.251)	0.992 (0.986–0.998)	3.172 (2.851–3.342)	0.094 (0.039–0.204)
	≥6	0.900 (0.817–0.950)	0.764 (0.757–0.768)	0.243 (0.197–0.289)	0.989 (0.982–0.996)	3.811 (3.361–4.096)	0.131 (0.065–0.242)
	≥7	0.867 (0.779–0.925)	0.811 (0.803–0.816)	0.279 (0.226–0.331)	0.986 (0.979–0.994)	4.578 (3.961–5.020)	0.164 (0.091–0.275)
	**≥8**	**0.811 (0.718–0.881)**	**0.845 (0.838–0.851)**	**0.307 (0.248–0.365)**	**0.982 (0.973–0.990)**	**5.245 (4.418–5.926)**	**0.223 (0.139–0.337)**
	≥9	0.778 (0.683–0.853)	0.871 (0.863–0.877)	0.337 (0.272–0.401)	0.979 (0.970–0.988)	6.014 (4.971–6.937)	0.255 (0.167–0.368)
	≥10	0.722 (0.625–0.804)	0.893 (0.885–0.900)	0.363 (0.293–0.434)	0.974 (0.964–0.984)	6.760 (5.435–8.049)	0.311 (0.217–0.424)
	≥11	0.700 (0.604–0.783)	0.917 (0.908–0.924)	0.414 (0.336–0.493)	0.973 (0.963–0.983)	8.392 (6.597–10.252)	0.327 (0.235–0.436)
AD	≥4	0.780 (0.723–0.829)	0.663 (0.650–0.675)	0.350 (0.307–0.392)	0.928 (0.909–0.948)	2.317 (2.069–2.549)	0.332 (0.254–0.425)
	**≥5**	**0.725 (0.667–0.777)**	**0.744 (0.731–0.757)**	**0.397 (0.349–0.445)**	**0.921 (0.902–0.940)**	**2.836 (2.478–3.192)**	**0.370 (0.295–0.456)**
	≥6	0.661 (0.602–0.715)	0.799 (0.785–0.811)	0.432 (0.379–0.486)	0.910 (0.891–0.930)	3.282 (2.801–3.792)	0.425 (0.351–0.507)
	≥7	0.606 (0.547–0.661)	0.842 (0.829–0.855)	0.471 (0.413–0.530)	0.902 (0.882–0.922)	3.842 (3.200–4.561)	0.468 (0.397–0.546)
	≥8	0.564 (0.508–0.618)	0.878 (0.864–0.890)	0.517 (0.453–0.580)	0.897 (0.877–0.916)	4.607 (3.744–5.622)	0.497 (0.429–0.570)
**GAD-2**							
GAD	≥2	0.933 (0.857–0.972)	0.686 (0.680–0.689)	0.200 (0.162–0.239)	0.992 (0.985–0.998)	2.973 (2.675–3.130)	0.097 (0.040–0.210)
	**≥3**	**0.756 (0.659–0.834)**	**0.876 (0.868–0.883)**	**0.340 (0.274–0.406)**	**0.977 (0.968–0.987)**	**6.107 (5.001–7.122)**	**0.279 (0.188–0.392)**
	≥4	0.600 (0.504–0.689)	0.931 (0.923–0.938)	0.422 (0.336–0.507)	0.965 (0.954–0.976)	8.651 (6.501–11.142)	0.430 (0.331–0.538)
AD	≥1	0.830 (0.777–0.874)	0.546 (0.534–0.557)	0.298 (0.262–0.335)	0.933 (0.912–0.954)	1.830 (1.666–1.972)	0.311 (0.226–0.418)
	**≥2**	**0.743 (0.686–0.794)**	**0.726 (0.713–0.738)**	**0.387 (0.340–0.433)**	**0.924 (0.905–0.943)**	**2.715 (2.388–3.035)**	**0.354 (0.278–0.441)**
	≥3	0.495 (0.441–0.548)	0.902 (0.889–0.914)	0.540 (0.471–0.610)	0.885 (0.865–0.905)	5.056 (3.982–6.386)	0.559 (0.495–0.629)

*AD, anxiety disorders; GAD, generalized anxiety disorder; PLR, positive likelihood ratio; NLR, negative likelihood ratio; PPV, positive predictive value; NPV, negative predictive value; CI, confidence interval. Recommended cut-off scores were indicated in bold*.

ROC analysis of the GAD-2 showed an AUC of 0.90 (95% CI: 0.87–0.93, SE = 0.02, *p* < 0.001), indicating high accuracy (33). At a cutoff score of 3 or greater, the balance of sensitivity and specificity reached its maximum. Sensitivity was 0.76 (95% CI: 0.66–0.83) and specificity was 0.88 (95% CI: 0.87–0.88) with a PPV of 0.34 (95% CI: 0.27–0.41), an NPV of 0.98 (95% CI: 0.97–0.99), a PLR of 6.11 (95% CI: 5.00–7.12), and an NLR of 0.28 (95% CI: 0.19–0.39) (Table 2). Both cutoff scores identified were consistent with previous meta-analysis of the GAD-7 and GAD-2 (17).

For identifying any anxiety disorder including GAD, ROC analysis of the GAD-7 revealed an AUC of 0.80 (95% CI: 0.76–0.83, SE = 0.02, *p* < 0.001), indicating moderate accuracy (33). At a cutoff score of 5 or greater, the balance of sensitivity and specificity reached its maximum. Sensitivity was 0.73 (95% CI: 0.67–0.78) and specificity was 0.74 (95% CI: 0.73–0.76) with a PPV of 0.40 (95% CI: 0.35–0.45), an NPV of 0.92 (95% CI: 0.90–0.94), a PLR of 2.84 (95% CI: 2.48–3.19), and an NLR of 0.37 (95% CI: 0.30–0.46) (Table 2).

ROC analysis of the GAD-2 showed an AUC of 0.78 (95% CI: 0.75–0.82, SE = 0.02, *p* < 0.001), indicating moderate accuracy (33). At a cutoff score of 2 or greater, the balance of sensitivity and specificity reached its maximum. Sensitivity was 0.74 (95% CI: 0.69–0.79) and specificity was 0.73 (95% CI: 0.71–0.74) with a PPV of 0.39 (95% CI: 0.34–0.43), an NPV of 0.92 (95% CI: 0.91–0.94), a PLR of 2.72 (95% CI: 2.39–3.04), and an NLR of 0.35 (95% CI: 0.28–0.44) (Table 2).

## Discussion

This study was conducted to determine whether the GAD-7 and GAD-2 were able to detect GAD specifically and any anxiety disorder including GAD. The results suggested that the Korean versions of the GAD-7 and GAD-2 are reliable and valid measures for detecting GAD. However, use of the GAD-7 and GAD-2 to screen for any anxiety disorder should be cautioned.

The GAD-7 and GAD-2 showed excellent internal consistency and good convergent validity with other anxiety measures. The total GAD-7 score was strongly correlated with the scores of the BAI and PSWQ. The total GAD-2 score, which was not statistically different from that of the GAD-7, was also significantly correlated with both BAI and PSWQ scores. These results mean that GAD-7 and GAD-2 have a good convergent validity with anxiety measures.

Both the GAD-7 and the GAD-2 were correlated with the depression scales. Specifically, the correlations between the GAD-7 and the depression measures were stronger than with the PSWQ. Correlations of the GAD-2 with the CES-D were higher than that of the PSWQ. High correlations between GAD-7/2 and depressive symptoms measures were not hypothesized, but interesting results since some of the previous studies reported similar correlational patterns (10, 34, 35). In addition, Watson (36) argued that GAD is more similar to depressive disorders than to the other anxiety disorders. More importantly, it has been reported that Asians with GAD and depressive disorders have more physical symptoms than cognitive symptoms (i.e., pathological worries) (8). Despite the high correlations between GAD-7/2 and depressive symptoms measures, participants with GAD had the highest means on the GAD-7/2 than those with other anxiety disorders or depressive disorders, providing evidence for discriminant validity of GAD-7/2, and their clinical utility as a screening tool for GAD. Therefore, after obtaining GAD-7 or GAD-2 results, clinicians should also gather additional information about depressive symptoms for differential diagnosis or treatment planning.

The Korean versions of the GAD-7 and GAD-2 detected GAD with excellent accuracy. ROC analysis showed high accuracy for both the GAD-7 and GAD-2 in detecting probable cases of GAD. These AUC values are relatively high compared with previous research (17). The optimal cutoff score for GAD, at which the balance of sensitivity and specificity was maximized, was 8 or greater for the GAD-7 and 3 or greater for the GAD-2. These cutoff points were consistent with the scores suggested by previous meta-analysis (17). Additionally, both the GAD-7 and GAD-2 showed low NPV, indicating a false negative rate of about 2% when detecting GAD with the GAD-7 and GAD-2. These characteristics indicate that the GAD-7 and GAD-2 are a useful screening tool for GAD patients in various settings. However, it should be noted that as in previous studies, PPV was quite low for detecting GAD using the GAD-7 or GAD-2 (10, 37, 38). The low PPV indicates that the GAD-7 and GAD-2 could detect too many false positives. At a cutoff score 8 or greater for the GAD-7, 69% of participants were not actual GAD patients, and at a cutoff score 3 or greater for the GAD-2, 66% of subjects were not actual GAD patients. This issue is partially due to the low prevalence of GAD (7.7% in this study) because PPV drops with a prevalence rate (33). We thus calculated PLR and NLR to compensate for the prevalence effects. PLR for the GAD-7 was 5.25, meaning that GAD-7 scores exceeding 8 are obtained approximately five times more often in subjects with GAD than subjects without GAD. PLR for the GAD-2 was 6.11, meaning that a GAD-2 score exceeding 3 is obtained approximately six times more often from subjects with GAD than subjects without GAD. These results indicate that both the GAD-7 and GAD-2 could provide “clinically useful information” in identifying GAD (33).

We also investigated whether the GAD-7 and GAD-2 could be used to detect any anxiety disorder. ROC analysis of the GAD-7 and GAD-2 indicated moderate accuracy; the cutoff score was 5 or greater for the GAD-7 and 2 or greater for the GAD-2. The GAD-7 cutoff score was quite lower than in previous meta-analysis (8 or greater) (17). In the case of the GAD-2, sensitivity and specificity varied throughout previous studies, and thus GAD-2 cutoff scores could not be drawn from the previous meta-analysis (17). Although NPV was high for both the GAD-7 and the GAD-2, PPV was quite low. Using the GAD-7 and GAD-2 cutoff scores, about 60% of subjects detected were not actual anxiety disorder patients. Moreover, the low PLR and high NLR were more problematic when detecting anxiety disorders using the GAD-7 or GAD-2. A PLR of < 3.00 and an NLR of more than 0.33 rarely alter clinical decisions (33), and thus the GAD-7 and GAD-2 do not provide additional information in detecting any anxiety disorders. Thus, to prevent misdiagnosis and unnecessary costly intervention when screening for any anxiety disorders, it is recommended that the GAD-7 or GAD-2 be used in combination with additional clinical interviews or other screening tools specifically designed to diagnose anxiety disorders (17).

The limitations of the current study are as follows. First, participants were not recruited by stratified random sampling. Although subjects were recruited randomly, with minimal exclusion criteria, from online advertisements and introduction by hospital staff, age and gender quotas were not applied. Many subjects of this study were women (66.7%), were in their 20s (42%), and were highly educated (an average of 14.63 years of education). Therefore, future study should be conducted with subjects with equal gender and age distribution. Second, it was unclear why the results of this study (low PLR and high NLR of the GAD-7 and GAD-2 in detecting anxiety disorders) differed from those of previous study (12). These discrepancies might be due to cultural factors. All of our subjects were Asian (i.e., South Korean), whereas about 97% of subjects in previous study reported white, Hispanic, and Black ethnic backgrounds. In a previous study, patients with anxiety disorders in Asia tend to report somatic symptoms as emotional distress (8). It is speculated that since the GAD-7 and GAD-2 items do not reflect or measure various somatic symptoms, GAD-7/2 in the current study might be poorer in identification of anxiety disorder in our study sample than previous studies. Cultural differences (or consideration) while administering and interpreting the GAD-7/2 scores have been reported in a previous study (39) in which Parkerson et al. (39) indicated that individuals who defined themselves as Black/African American endorsed significantly lower on some items (e.g., feeling nervous, irritable, restless, etc.) of the GAD-7 than other ethnic (i.e., White and Hispanic) group. Thus, these discrepancies, which are not yet fully understood, should be a subject of future study.

Despite these limitations, the current study provides evidence on the psychometric properties and clinical utility of both the GAD-7 and the GAD-2 as reliable and valid screening tools for people with GAD. Because the GAD-2 is an ultra-brief measurement, it can be a useful tool for various clinical settings (e.g., primary care) with limits on time and resources. It is expected that both measures could be widely used to detect GAD in many clinical settings, and thus provide optimal and timely intervention in community.

## Data Availability

The datasets generated for this study are available on request to the corresponding author.

## Ethics Statement

This study was carried out in accordance with the recommendations of Korea University Institutional Review Board [1040548-KU-IRB-15-92-A-1(R-A-1)(R-A-2)(R-A-2)] with written informed consent from all subjects. All subjects gave written informed consent in accordance with the Declaration of Helsinki. The protocol was approved by the Korea University Institutional Review Board [1040548-KU-IRB-15-92-A-1(R-A-1)(R-A-2)(R-A-2)].

## Author Contributions

J-KA interpreted the results and drafted the manuscript. J-KA and YK analyzed data. YK collected data, and drafted introduction and method. K-HC designed the study, supervised clinical and research assistants, and reviewed manuscript. All the authors approved the final manuscript and significantly contributed to the current study.

### Conflict of Interest Statement

The authors declare that the research was conducted in the absence of any commercial or financial relationships that could be construed as a potential conflict of interest.
